# Is there hierarchical generalization in response-effect learning?

**DOI:** 10.1007/s00221-022-06473-w

**Published:** 2022-11-17

**Authors:** Lea Eichfelder, Volker H. Franz, Markus Janczyk

**Affiliations:** 1 Department of Psychology, University of Bremen, Bremen, Germany; 2 Department of Computer Science, University of Tübingen, Tübingen, Germany

**Keywords:** Ideomotor theory, Response-effect learning, Response-effect compatibility, Generalization, Superordinate category

## Abstract

Ideomotor theory is an influential approach to understand goal-directed behavior. In this framework, response-effect (R-E) learning is assumed as a prerequisite for voluntary action: Once associations between motor actions and their effects in the environment have been formed, the anticipation of these effects will automatically activate the associated motor pattern. R-E learning is typically investigated with (induction) experiments that comprise an acquisition phase, where R-E associations are presumably learned, and a subsequent test phase, where the previous effects serve as stimuli for a response. While most studies used stimuli in the test phase that were identical to the effects in the acquisition phase, one study reported generalization from exemplars to their superordinate category (Hommel et al., Vis Cogn 10:965–986, 2003, Exp. 1). However, studies on so-called R-E compatibility did not report such generalization. We aimed to conceptually replicate Experiment 1 of Hommel et al. (Vis Cogn 10:965–986, 2003) with a free-choice test phase. While we did observe effects consistent with R-E learning when the effects in the acquisition phase were identical to the stimuli in the test phase, we did not observe evidence for generalization. We discuss this with regard to recent studies suggesting that individual response biases might rather reflect rapidly inferred propositional knowledge instead of learned R-E associations.

## Introduction

Human actions aim to attain goals and to change the environment in desired ways. The resultant changes and consequences are often referred to as action effects. Goal-driven behavior thus requires an actor to know what (motor) behavior leads to which consequence(s) in the environment. The present study investigates one particular question on learning associations between actions and their effects, namely whether the effects’ representations generalize to a higher hierarchy level.

### Ideomotor theory

Ideomotor theory is a general framework of action control (e.g., Harleß 1861; James 1890) that highlights the role of action effects. More precisely, selecting an action is achieved by an anticipation of the effects of the to-be-produced action, or more broadly, intended environmental changes. Having its roots in philosophical ideas of the nineteenth century (see also Janczyk and Kunde 2020; Pfister and Janczyk 2012; Stock and Stock 2004), this theory was re-discovered in the twentieth century by Greenwald (1970) and has influenced psychological experimental research on human action since then. Following this line of thought, several authors (e.g., Elsner and Hommel 2001) distinguished two (sequential) stages: Stage 1, during which bidirectional associations between actions and their subsequent effects are established, and Stage 2, in which the acquired action-effect relations are used for planning and executing actions. In this latter stage, mental anticipation of the desired effect is assumed to activate the respective motor patterns required for its achievement.

### Evidence for response-effect learning and anticipation

The acquisition of associations between actions and their effects (i.e., Stage 1) has been addressed with an experimental approach that has sometimes been termed induction experiments (Paelecke and Kunde 2007). The general approach of such studies is to first associate bodily movements with arbitrary, but contingent effects in an acquisition phase. Thus, participants perform bodily movements (e.g., keypresses) that lead to certain effects during this phase. The repetition of these movements and the contingent occurrence of the effects is thought to yield bidirectional associations. In a subsequent test phase, these associations are then tested for, with the former effects now serving as response eliciting stimuli.

An influential study with this method was conducted by Elsner and Hommel (2001). During the acquisition phase, participants were to choose their motor response freely between two response options (in this case, a left versus right keypress), both triggering a contingent effect (in this case, a low- versus high-pitch tone). During 200 valid acquisition phase trials, bidirectional associations between the responses and their effects were expected to be formed. To empirically assess the occurrence of response-effect (R-E) learning, the former action effects subsequently served as imperative stimuli during a test phase, with either a forced-choice (Exp. 1) or a free-choice task (Exp. 2–4). In Experiment 1, reaction times (RTs) and percentages of error were measured. Performance was better if a stimulus required the response that previously produced this very stimulus as the effect (“non-reversal”-group) compared with when the stimulus now required the other response (“reversal”-group). In Experiments 2–4, the percentage of acquisition-congruent choices served as the main dependent variable. The observation of a significant response bias (i.e., more than 50% congruent choices) then points towards successful R-E learning. This result has been replicated directly (Janczyk et al. 2022, Exp. 3) as well as conceptually (e.g., Vogel et al. 2018; Watson et al. 2015, Exp. 1; Pfister et al. 2011; Wolfensteller and Ruge 2011). On the other hand, when Watson et al. (2015) used a more complex version of the standard experiment (i.e., four [instead of two] effects with two or four responses) in their Experiment 2, the reported lack of a response bias points at possible limitations of R-E learning.

Evidence for Stage 2, that is, when acquired R-E associations are used for planning and execution of actions, comes mostly from R-E compatibility experiments (Kunde 2001; see also Földes et al. 2018; Koch and Kunde 2002; Kunde 2003; Janczyk et al. 2015, 2017, as further examples). For example, in Experiment 1 of Kunde (2001), participants had to respond to an imperative stimulus (a color patch) by pressing one of four horizontally aligned keys. Each of the four possible responses triggered the illumination of one of four spatially corresponding, horizontally aligned boxes on a screen. In separate blocks, the keypresses either led predictably to a spatially compatible effect (e.g., far-left keypress → the box on the far-left screen side is illuminated) or predictably to a spatially incompatible effect (e.g., far-left keypress ﻿→  a box two positions adjacent of the corresponding box is illuminated). An R-E compatibility effect was observed in that participants responded faster and more accurately when the (anticipated) effect and the response were spatially compatible than when they were incompatible. This result has been interpreted to mean that the anticipated effects prime compatible responses (Janczyk and Lerche 2019).

### Generalization (and abstraction) of action effects

Most induction experiments used response-eliciting stimuli in the test phase that were physically identical to the effects in the acquisition phase. However, Hommel et al. (2003) argued that, in everyday life, an action typically does not elicit the exact same effects. They, therefore, investigated in Experiment 1 whether R-E associations and the respective representation of the action effects generalize to their superordinate categories (Rosch et al. 1976). More precisely, one group of participants (“category group”) received the category words “furniture” and “animal” (in Spanish) as action effects during the acquisition phase, while another group (“exemplar group”) received the corresponding exemplar words “chair” and “dog”. In a forced-choice test phase, only the category words were presented as stimuli for both groups. The respective compatibility effect for RTs was of the same size for both groups, pointing to a generalization of action effects in the exemplar group to other, related stimuli, in this case to the superordinate categories. In two further experiments, the authors reported that such generalization of R-E learning is not restricted to category labels, but transfers to other category members as well (Exp. 2), and that the transfer of R-E associations is not even restricted to members of the same category, but can also be mediated by perceptual features (Exp. 3).

In contrast, studies on R-E compatibility yielded little evidence for an abstraction or generalization of action effects. Koch and Kunde (2002) conducted two experiments in which participants had to utter color words (e.g., “blue” or “green”) as a response, followed by the written color words as visual action effects. The resulting R-E compatibility effect was larger when the visual effect (the color word) was also written in the respective color (e.g., “blue” written in blue), compared to a control group that received color words written in white letters. This result seems to point towards the occurrence of abstraction from the verbal response to the visual color effect. On the other hand, taking into account that reading a color word might result automatically in phonological recoding, this could be either compatible or incompatible with the verbal response as well. To investigate this possibility, Földes et al. (2018) conducted an experiment, in which the verbal response and the auditory effect in a bilingual condition had no phonological overlap. For example, if the response was to be given in German (e.g., “Schwein”), the English translation (e.g., “pig”) was used as an action effect. In a monolingual (control) condition with phonological overlap, an R-E compatibility effect was observed, while in the bilingual condition, this was not the case. Moreover, a recent study (Koch et al. 2021) did not report an R-E compatibility effect when they used the category words “animals” and “furniture” in German as responses and either the same category words or exemplars of these categories (“horse” and “chair” in German) as effects (similar to what has been used by Hommel et al. 2003, Exp. 1).

### The present experiment

Given this mixed evidence on abstraction and generalization of action effects, we re-assessed the observation reported by Hommel et al. (2003, Exp. 1). To this end, we ran an experiment with the same acquisition phase as in the original experiment: the *category* *group*, which served as a control group, produced category words as effects, while the *exemplar group*, that is, our experimental group, produced exemplar words. However, instead of a forced-choice test phase with RTs as the dependent variable, we employed a free-choice test phase with the percentage of congruent choices as the dependent variable to assess whether a response bias exists. This choice was motivated by arguments that R-E learning occurs only during free-choice tasks (e.g., Herwig et al. 2007) and that, more importantly, R-E associations express themselves more readily in behavior if the test phase involves a free-choice task (Pfister et al. 2011). Thus, using a free-choice test phase should be particularly suited to detect signs of R-E learning.

The test phase comprised 50% go trials, in which a category word was presented as the stimulus, and 50% no-go trials, in which a letter string without semantic meaning (“XXXXX”) was presented. Randomly intermixing these trials aimed to prevent participants from pre-planning their response prior to the onset of each stimulus (see Elsner and Hommel 2001, Exp. 3).

The main question of this experiment is whether action effects, associated with bodily movements, generalize to their superordinate category (e.g., whether learning an association between a response and the exemplar word “chair” generalizes to its category “furniture”, such that it is also associated with the corresponding response). Similar to Experiment 1 of Hommel et al. (2003), the category group received category words as effects. When these words are then presented as stimuli, we expected to observe a response bias in the test phase, replicating the basic R-E learning effect. A similar response bias in the exemplar group (which received exemplar words during the acquisition phase) would indicate generalization. A smaller or no response bias in this group would indicate that less or no generalization has occurred, respectively.

## Method

### Open practices statement

This study was preregistered on AsPredicted.org before data collection. The pre-registration is available at https://aspredicted.org/g8gu4.pdf. Data are available publicly at https://osf.io/z3qc4/.

### Participants

The preregistered sequential sampling plan resulted in *N* = 100 participants (mean age = 25.43 years, 77 females, 23 males, 0 non-binary) to be included in the analysis. In total, 99 students from the University of Bremen participated for course credit and 6 other people from the Bremen area participated without any reimbursement. Five participants were excluded from further analyses due to the exclusion criteria (see below). All participants were either native German speakers or had advanced written and spoken knowledge of German and had normal or corrected-to-normal vision. Eighty-eight participants were right-handed, 12 left-handed, and 0 ambidextrous. All participants were naïve to the hypotheses of this experiment.

The following, pre-registered exclusion criteria were applied: Participants who did not press the left and the right key at least 80 times each (out of a maximum of 100) during the acquisition phase were excluded from further analyses (see Hommel et al. 2003, Exp.1, for this criterion). This led to the exclusion of three participants. Additionally, two participants who responded in more than 20% of the no-go trials were excluded. The sample size was determined by using Bayesian sequential sampling based on Bayes factors (BFs) with the following stopping rules (see Schönbrodt and Wagenmakers 2018, and Schönbrodt et al. 2017, for threshold determination), according to which sampling would be stopped when one of the following conditions was met:

1. A BF_10_ < 1/10 was calculated for the one-sample Bayesian *t* test of the category group. This result would mean that participants have reacted randomly during the test phase instead of showing a response bias, in turn suggesting that no learning of associations at all took place during the acquisition phase of the experiment even in the category group.
2. A BF_10_ ≥ 6 was calculated for the one-sample Bayesian *t* test of the category group (suggesting that learning took place for this group) and at the same time a BF_10_ of ≥ 6 or < 1/6 was calculated for the two-sample *t* test comparing the category and the exemplar group. The first result would mean that no, or at least no full, generalization occurred. The second result would mean that full generalization occurred, as the response biases would be of the same size in both groups. In the first case, a one-sample Bayesian *t* test would also be calculated for the exemplar group to assess whether some signs of generalization can be observed, as would be indicated by a response bias in this group (and a corresponding BF_10_ ≥ 1).
3. A maximum number of *n* = 50 participants per group has been reached.

BFs were monitored after data from an initial sample of 20 participants per group have been collected. Then, they were monitored after four additional participants (the smallest number required to include all counterbalancing variables). More precisely, the participants were each assigned to one of four conditions resulting from the combination of the group (category group vs. exemplar group) and R-E mapping during the acquisition phase within each group (thus *n* = 25 participants per condition in the final sample) counterbalanced across participants. All participants took part in the experiment in a single session of approximately 35 min.

### Stimuli and apparatus

Stimulus presentation and response collection were done via a standard PC connected to a 17-inch CRT monitor. The category words “FURNITURE” and “ANIMALS” in German and the exemplar words “CHAIR” and “CAT” in German served as effects and stimuli. They were written in white color against a black background in capital letters, with a height of approximately 1 cm. The “D” and the “L” key of a standard QWERTZ keyboard served as left and right response keys, respectively, and the spacebar served as the response key in catch trials.

### Task and procedure

**Fig. 1:**
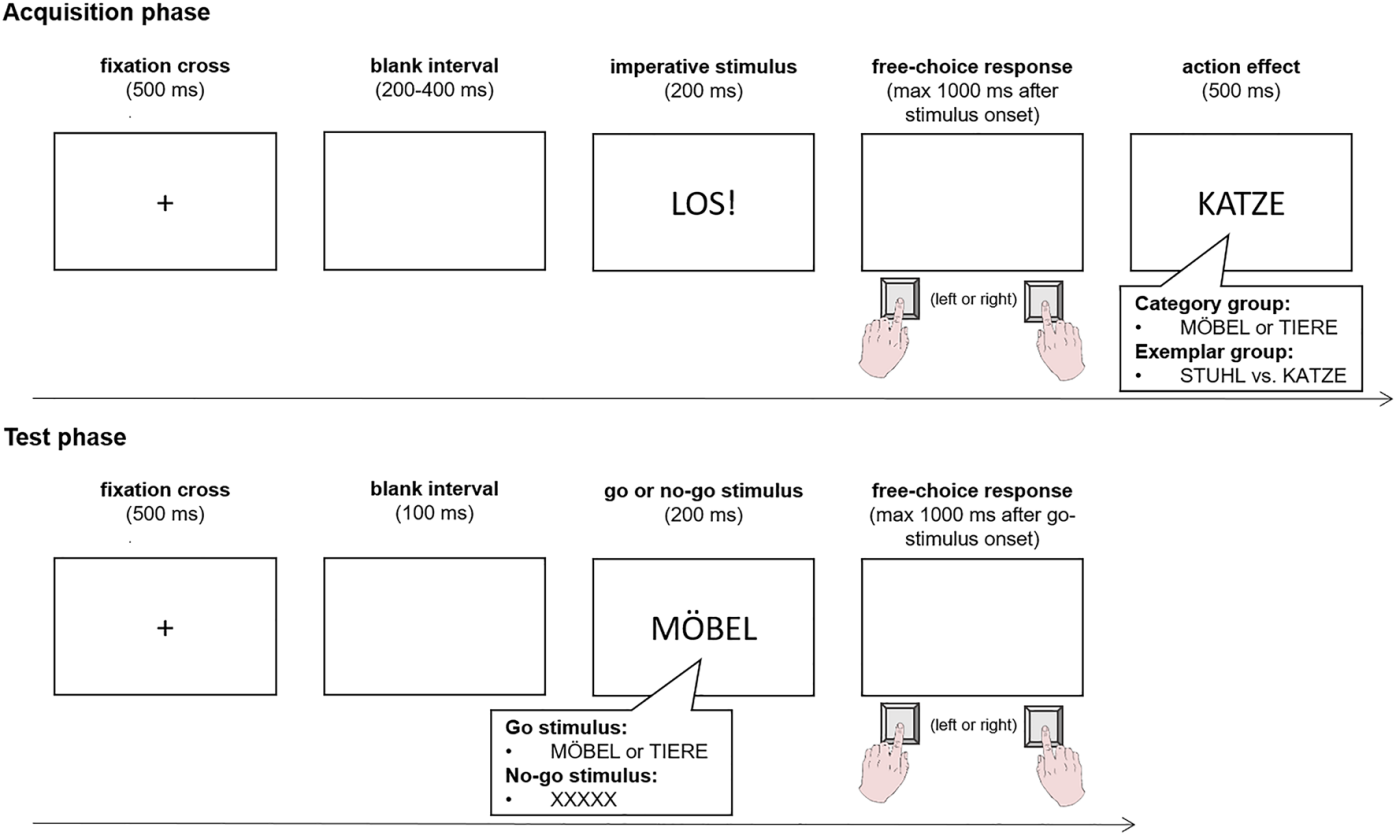
The upper panel illustrates the trial sequence and design of the acquisition phase, while the lower panel illustrates the trial sequence and design of the test phase. Stimulus words are presented here in the German language as in the experiment proper (translation: LOS! = GO!, MÖBEL = FURNITURE, TIERE = ANIMALS, KATZE = CAT, STUHL = CHAIR)

Testing took place in dimly lit, sound-attenuated experimental cabins. The trial sequence of the *acquisition phase* is visualized in the upper panel of Fig. 1. Each trial began with the presentation of a white fixation cross in the screen center for 500 ms, followed by a blank interval with a randomly determined length between 200 and 400 ms. After that, the stimulus (the German word “LOS!” written in white letters) was presented in the screen center for 200 ms, indicating the participants to press the left or right key as fast as possible within 1000 ms. Each key produced a different visual effect, depending on the group and R-E mapping (see Table 1). The effect words were fully contingent on the identity of the preceding response so that an acquisition of stable R-E associations is favored.

**Table 1:** R-E mapping-dependent relations of responses and their effects (R-E) during the acquisition phase for both groups and stimuli of the test phase (with XXXXX being the no-go stimulus)

Group	Acquisition phase			Test phase
	Counterbalanced R-E mapping	Response	Effect	Stimuli
Category	1	Left	FURNITURE	FURNITURE ANIMALS XXXXX
		Right	ANIMALS	
	2	Left	ANIMALS	
		Right	FURNITURE	
Exemplar	1	Left	CHAIR	
		Right	CAT	
	2	Left	CAT	
		Right	CHAIR	

The stimuli for catch trials in the acquisition phase were FRUIT and APPLE for the category and the exemplar group, respectively. Note that all stimuli in the actual experiment were in the German language

In the *category group*, these effect words were the category words. In the *exemplar group*, the effect words were the corresponding exemplar words. In each group, the R-E mapping was counterbalanced (see also Table 1 for a complete overview). To be consistent with Experiment 1 of Hommel et al. (2003), we also included catch trials in 5% of the acquisition phase trials. In those catch trials, the catch words “FRUIT” (category group) or “APPLE” (exemplar group) in German appeared instead of the regular effect words. These catch trials were presented at random positions within the acquisition phase and participants had to respond as fast as possible by pressing the space bar within 2000 ms. Errors in catch trials were fed back to participants by displaying an error message for 500 ms in the screen center (“please react faster by pressing the SPACE BAR” in the German language). Trials with RTs longer than 1000 ms were considered omissions while RTs shorter than 100 ms were considered anticipation errors, and both were fed back to the participants by displaying an error message for 500 ms in the screen center (“too fast!” and “too slow!” in the German language, respectively). These trials were repeated at a random position of the block. Each trial ended with an intertrial interval of 2000 ms before the next trial started.

After having finished 200 valid acquisition phase trials, the *test phase* commenced. The trial sequence of the test phase is visualized in the lower panel of Fig. 1. Each trial started with the presentation of a fixation cross for 500 ms in the screen center, followed by a blank interval (100 ms). After that, in half of the trials, the imperative go-stimulus (i.e., one of the two category words, both equally often) appeared for 200 ms on the screen and required a left or right response in a free-choice task within 1000 ms. In the other half of trials, the letter string “XXXXX” appeared in the screen center as a no-go stimulus and participants had to withhold any response (for a maximum of 2000 ms). All erroneous trials (anticipations, omissions, as well as responses in no-go-trials) were fed back to the participants by displaying an error message for 500 ms and were repeated at a random position of the block. All stimuli were intermixed randomly.

Participants were first instructed about the acquisition phase, both with written instructions presented on the computer screen and verbally by the experimenter. Once the acquisition phase has ended, they received the corresponding instructions for the test phase. With regard to the free-choice task, participants were instructed to choose freely between both response keys, but to press them about equally often and to avoid response patterns like alternating both keys.

### Design and analyses

Trials with anticipations (RT < 100 ms) and omissions (RT > 1000 ms) were excluded. Thus, each participant contributed 200 valid acquisition trials and 200 valid test trials. As described in our pre-registration, we used Bayesian sequential sampling (see also “participants” for details). For this, we calculated one-sample Bayesian *t* tests for the category group and for the exemplar group and compared the percentage of congruent choices in each group against a chance level of 50%. A two-sample Bayesian *t* test was calculated for the group comparison. All corresponding BF_10_ were calculated using the R-package BayesFactor (Morey et al. 2022; using the default settings of a Cauchy prior on the standardized effect size with the scale parameter set to $\frac{\surd 2}{2}$ and a noninformative Jeffreys prior on the variance). A BF_10_ > 1 supports the alternative hypothesis, while 0 < BF_10_ < 1 supports the null hypothesis. To allow for an easier comparison to traditional methods, we also provide the corresponding frequentist *t* tests. For our data, both approaches yielded similar results.

## Results

### Acquisition phase

Anticipations, omissions, and missed catch trials (i.e., those with an RT > 2000 ms) were recorded in 2.59, 4.70, and in 0.88% of all trials, respectively, and these trials were excluded from analyses. Both response keys were used about equally often (left key: average of 99.96 times per participant, right key: average of 100.04 times per participant). Biases during the acquisition phase were calculated by dividing the number of left responses by the number of right responses for each participant, and they ranged from 0.71 to 1.33.

Mean RTs were 380 ms for the category group and 403 ms for the exemplar group. Bayesian as well as frequentist two-sample *t* tests indicate that those values are not reliably different, BF_10_ = 0.54, *t*(98) = − 1.46, *p* = 0.148, *d* = − 0.29.

### Test phase

**Fig. 2:**
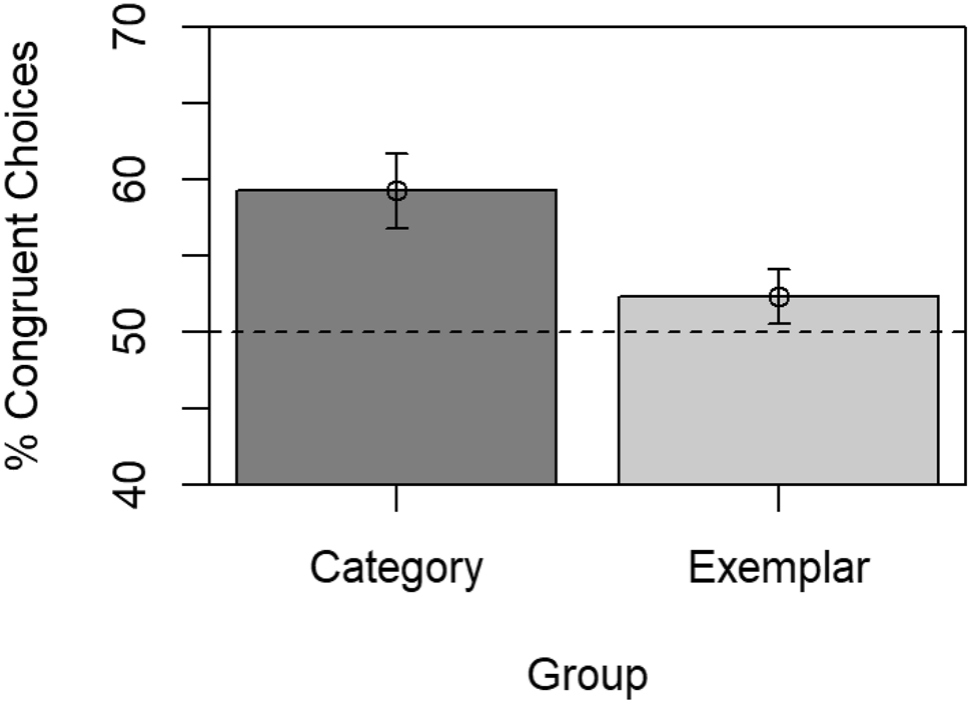
Mean percentage of congruent choices in the category and the exemplar group. The dashed horizontal line at 50% indicates the expected value when response choice was entirely random and the error bars are the standard errors of the means

Anticipations, omissions, and false alarms in no-go trials were recorded in 0.01, 0.47, and 2.35% of all trials, and these trials were excluded from analyses. Mean RTs in go trials were 433 ms in the category group and 413 ms in the exemplar group, and were not reliably different, BF_10_ = 0.85, *t*(98) = 1.78, *p* = 0.078, *d* = 0.36. The percentage of congruent choices was then calculated for each participant and used as the dependent variable in the following analyses. Figure 2 illustrates the means of these percentages per group.

For the category group, inspection of Fig. 2 suggests that the percentage of congruent choices clearly deviates from chance level of 50%, thus indicating a response bias. This is supported by statistical tests, with evidence for the alternative hypothesis of a response bias, BF_10_ = 48.01, *t*(49) = 3.69, *p* = 0.001, *d* = 0.52.^1^ Thus, an R-E association seems to have been learned during the acquisition phase in this group.

For the exemplar group, however, an inspection of Fig. 2 shows that the percentage of congruent choices is smaller than for the category group, and the comparison of both groups yields some evidence toward the alternative hypothesis of a group difference, BF_10_ = 1.95, with the frequentist *t* test indicating a significant difference, *t*(98) = 2.25, *p* = 0.027, *d* = 0.45. Indeed, the percentage of congruent choices in the exemplar group is around chance-level, and the statistical tests favor the null hypothesis of chance-level performance, BF_10_ = 0.32, *t*(49) = 1.26, *p* = 0.212, *d* = 0.18. These results suggest the absence of a response bias in the exemplar group.

Taken together, evidence for (a full) generalization in the exemplar group seems weak at best. If at all, the evidence seems to favor the null hypothesis of chance-level performance in the exemplar group.

## Discussion

The present study set out to re-assess generalization (from exemplars to categories) in R-E learning. Evidence for generalization was reported by Hommel et al. (2003) in a series of three induction experiments (Elsner and Hommel 2001), while recent research on R-E compatibility did not reveal evidence for an abstraction or generalization of action effects (Földes et al. 2018; Koch et al. 2021). Given this mixed evidence, our aim was to investigate whether generalization could be observed in a conceptual replication of Hommel et al.’s (2003) Experiment 1. In contrast to the original experiment, however, we employed a free-choice test phase which is considered more sensitive than a forced-choice task. Results of our experiment do not support generalization though. More precisely, while we did observe a response bias in the category group, we did not observe evidence for generalization from exemplars to their superordinate categories in our exemplar group. Rather the results are more in line with the conclusion that no full or even no generalization at all occurred in the exemplar group and R-E associations are bound to the exact stimuli as were presented during acquisition.

A limitation of the present results is the strength of evidence as indicated by the respective BFs. According to the suggestions of Jeffreys (1961),^2^ we have obtained strong evidence for a response bias in the category group. In contrast, the evidence for the alternative hypothesis of a group difference resides in an inconclusive range, although it was still in the direction of evidence toward the alternative hypothesis. In addition, the evidence for the null hypothesis of no response bias in the exemplar group just crossed the border from inconclusive to substantial evidence. Despite this, we believe the overall results still support the conclusion of no (or only little) generalization from exemplars to categories. Note also that the frequentist *t* tests do not contradict the BFs, but rather are well in line with this conclusion.

What could be the reasons for the diverging results in the literature? Of course, either our results or those reported by Hommel et al. (2003) could represent a chance finding. Yet, instead of dismissing one or the other set of results as invalid at present, we suggest considering design differences instead. A crucial difference is that we used a free-choice test phase and the percentage of congruent choices as the dependent variable, while Hommel et al. used a forced-choice test phase and analyzed RTs and the percentages of error. There are a number of reasons to consider free-choice tasks, and thus the response bias measured with them, as a more sensitive measure to assess the existence of acquired R-E associations compared with the forced-choice task used by Hommel et al. For example, Herwig et al. (2007) argued that R-E associations are not even acquired in forced-choice, but only in free-choice tasks, as only the latter are thought to operationalize intention-based action control (but see Naefgen et al. 2018; Naefgen and Janczyk 2018, for a critical view on this). Yet, Pfister et al. (2011) demonstrated that R-E associations are acquired regardless of the acquisition phase task if the test phase involves a free-choice task. Thus, acquired R-E associations might be more easily expressed in free-choice tasks. Despite this, no signs of generalization from the exemplar to the category level were observed.

In sum, we aimed at demonstrating evidence for generalization in the exemplar group, but rather failed to do so. In light of this, in the following, we will discuss our results against the background of a recent study. Specifically, Sun et al. (2020, Exp. 1) conducted an induction experiment with a free-choice test phase as well. To measure the influence of task instructions (see below) on individual response strategies, participants answered a questionnaire after the test phase to assess their knowledge about the acquired R-E relations. Participants were indeed largely able to report the acquired relations correctly, and this was irrespective of whether participants received detailed information on the R-E relations with the instructions or not, and a response bias was observed in both groups as well. When inspecting individual data, however, it appeared as if the (overall) response bias in Experiment 1 of Sun et al. on the group level was driven by a small number of participants with very large response biases, while the majority responded at or close to chance level (thus not showing a response bias), yielding a bimodal distribution of the percentages of congruent choices. Sun et al. reasoned that a free-choice task in the test phase – compared to a forced-choice task – does allow for deliberate response strategies and the observed bimodal distribution in the free-choice task does, therefore, “not provide strong evidence for the automatic nature of this effect” (p. 7).^3^ Rather, it seems as if some participants opted deliberately to “respond in line with the mapping learned in the acquisition phase” (p. 7).

That response strategies indeed play a role in free-choice tasks was also investigated by Vogel et al. (2018) who focused on individual differences in this regard by using a free-choice mouse tracking task. The authors identified two main groups of participants: one group chose their response before or at the beginning of each trial (i.e., prior to stimulus onset) and the decision was not affected by stimulus identity. The second group chose their response during the trial (i.e., after stimulus onset) and the choice was affected by stimulus identity. These differences in decision strategies can influence the results of free-choice tasks, as only the second group showed an impact of the R-E association acquired during the acquisition phase on the response behavior. Thus, these results also suggest that care is needed when interpreting the response bias in free-choice experiments.

**Fig. 3:**
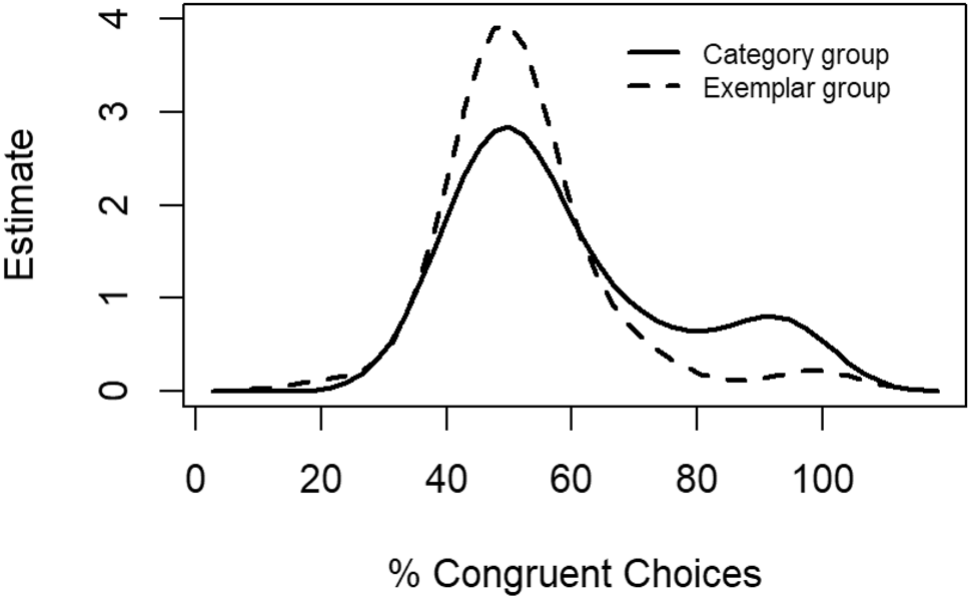
Kernel density plots of the response biases separately for the category group and the exemplar group

Against this background, we also explored the distribution of the percentages of congruent choices separately for our category and exemplar groups (see Fig. 3). Indeed, the distribution of the response bias in the category group points towards a bimodal distribution: In both groups a large number of participants has chosen their responses randomly, as the percentages of congruent choices revolve around 50%. Yet, there are few participants with very high individual response biases as well, and this is particularly the case in the category group. This impression was corroborated by calculating the bimodality coefficient for both groups (BC; SAS Institute Inc. 1990; see also Freeman and Dale 2013; Pfister et al. 2013) and comparing it to a value of BC_crit_ = 0.55. Higher values point towards a bimodal distribution, whereas lower values point towards a unimodal distribution. Using the procedures of Kieslich et al. (2022), the BCs were 0.65 and 0.50 for the category and the exemplar group, respectively. These results indeed suggest a bimodal distribution in the category group.

Thus, in our category group, the observed response bias seems to be driven by few participants (as in Exp. 1 of Sun et al. 2020) that applied deliberate response strategies during the test phase and not by the former action effects (automatically) triggering the acquisition-congruent actions during the test phase via bidirectional associations, as suggested in the literature on R-E Learning (e.g., Elsner and Hommel 2001; Hommel et al. 2003; see also Moeller and Pfister 2022). The question arises though, why a bimodal distribution did not occur in our exemplar group. Apparently, it makes a difference if the effects of the acquisition phase and the stimuli of the test phase are physically identical, at least for participants who seem to apply deliberate response strategies during the test phase. For those participants, the possibility to use a strategy such as responding in line with the learned R-E mapping might have become more obvious than for those in the exemplar group, for whom the action effects and test phase stimuli differed semantically and perceptually.

This interpretation would also point to a lack of generalization in the exemplar group, although it is fair to ask, what exactly is learned and expressed by participants in experiments like ours, that is, when using a free-choice test phase? According to Sun et al. (2020), the response bias obtained in their Experiment 1 was not caused by ‘modal’ R-E associations that had developed during the acquisition phase, but instead by more ‘amodal’, propositional, and spontaneously inferred propositional knowledge. The terms modal and amodal here are used akin to the taxonomy of abstraction proposed by Reed (2016) according to which modal refers to a concrete (‘sensory’) representation, while amodal refers to an abstract (‘linguistic’) representation instead. In the case of automatic associations as typically assumed in R-E learning research, associations between stimulus-/effect- and response-representations are learned, likely outside of awareness, and activating one representation automatically activates the other one. With propositional knowledge, as suggested also by Sun et al. (2020), we mean that participants explicitly learn rules about the co-occurrence of particular effects/stimuli and responses and might (or might not) rely on them to choose responses in the test phase. Notably, Mitchell et al. (2009) have argued for a propositional account of putative associative learning in a broader context as well. At this point, we cannot make a definite statement regarding the nature of what has been learned in our experiment (and in previous experiments as well). However, the reasoning of Sun et al. and the results presented here might be taken to question the usefulness of individual response biases as a measure to quantify (modal) R-E learning in a valid way.

## Conclusion

In sum, the results from our experiment as well as results from R-E compatibility experiments (Földes et al. 2018; Koch et al. 2021) contrast with the generalization reported by Hommel et al. (2003). Before questioning this latter study’s results though, it seems worthwhile to consider design choices more thoroughly. In particular, as recent literature suggests (Sun et al. 2020, 2022), the percentages of congruent choices and a resultant response bias as measured with free-choice test phases is possibly more reflecting propositional knowledge that is inferred by the participants, rather than an association of particular responses and their effects. This possibility should be considered in future research.

## Data Availability

Data are available at https://osf.io/z3qc4/.

## Footnotes

1 Note that applying one-tailed *t* tests to test directed hypotheses here and in the following would not change the results qualitatively.

2 Jeffreys (1961) interprets BF_10_ < 1/10 as strong evidence for H_0_, BF_10_ between 1/10 and 1/3 as substantial evidence for H_0_, BF_10_ between 1/3 and 3 as inconclusive results, BF_10_ between 3 and 10 as substantial evidence for H_1_, and BF_10_ > 10 as strong evidence for H_1_.

3 If acquisition of R-E associations is automatic and if the stimuli further automatically induce a response tendency toward the associated response, the distribution of the percentage of congruent choices would be expected unimodal with the peak shifted in the direction of > 50%.
